# Successful Treatment of a Child With Epileptic Encephalopathy With Spike-Wave Activation in Sleep and GRIN2A Variant Using Sulthiame

**DOI:** 10.7759/cureus.34686

**Published:** 2023-02-06

**Authors:** Joana Pereira-Nunes, José Maria Sousa, Jacinta Fonseca, Cláudia Melo, Dílio Alves, Mafalda Sampaio, Raquel Sousa

**Affiliations:** 1 Department of Pediatrics, Centro Hospitalar Universitário de São João, Porto, PRT; 2 Department of Gynecology-Obstetrics and Pediatrics, Faculty of Medicine of Porto University, Porto, PRT; 3 Department of Neuroradiology, Centro Hospitalar Universitário de São João, Porto, PRT; 4 Pediatric Neurology Unit, Department of Pediatrics, Centro Hospitalar Universitário de São João, Porto, PRT; 5 Department of Neurophysiology, Centro Hospitalar Universitário de São João, Porto, PRT

**Keywords:** sulthiame, nmda receptor, grin2a variant, glun2a protein, corticosteroids

## Abstract

Epileptic encephalopathy with spike-wave activation in sleep (EE-SWAS) and developmental EE-SWAS (DEE-SWAS) are characterized by variable combinations of cognitive, language, behavioral, and/or motor regression associated with continuous or near-continuous diffuse spike-and-wave complexes during sleep. Glutamate ionotropic receptor NMDA type subunit 2A (*GRIN2A*) variants have been associated with EE-SWAS. It encodes the most relevant GluN2 subunit of the N-methyl-D-aspartate receptor (NMDAR). Sulthiame reduces NMDAR-mediated neuronal excitability and has been progressively used as monotherapy in self-limited epilepsy with centrotemporal spikes (SeLECTS) or as add-ontherapy in EE-SWAS/DEE-SWAS. A five-year-old female, with family history of epilepsy, was initially diagnosed with SeLECTS and medicated with valproic acid (VPA). One year later, she presented a focal to bilateral tonic-clonic seizure during sleep and learning difficulty. The electroencephalogram revealed continuous spike-and-wave during sleep leading to the diagnosis of EE-SWAS. Prednisolone was effective, but there was repeated recurrence after its discontinuation and associated adverse effects. As an alternative, sulthiame was added to VPA. Four years later, she remains clinically stable. Genetic testing revealed a *GRIN2A *missense variant, C.3228C>A (p.Asn1076Lys). Sulthiame appeared effective in this recurrent EE-SWAS child, who presented a *GRIN2A *missense variant with possible NMDAR gain-of-function and adverse effects of corticosteroids. Functional studies​​​​​​​ of *GRIN2A *variants might become a future tool for individualized therapies.

## Introduction

Epileptic encephalopathy with spike-wave activation in sleep (EE-SWAS) and developmental EE-SWAS (DEE-SWAS) are a spectrum of rare conditions characterized by a variable combination of cognitive, language, behavioral, and motor regression in association with marked activation of epileptiform sleep abnormalities, namely continuous or near-continuous diffuse spike-and-wave complexes during sleep [1]. DEE-SWAS occurs in patients with preexisting neurodevelopmental disorders [1]. These designations recently replaced the former epileptic encephalopathy with continuous spike-and-wave during sleep (ECSWS) and include Landau-Kleffner syndrome (LKS), the two most severe epilepsy-aphasia syndromes (EAS) entities [1,2]. Self-limited epilepsy with centrotemporal spikes (SeLECTS), previously known as benign childhood epilepsy with centrotemporal spikes (BECTS) or rolandic epilepsy (RE), was considered a mild form of EAS and may evolve into EE-SWAS or DEE-SWAS [1,2].

Glutamate ionotropic receptor NMDA type subunit 2A (*GRIN2A*) gene variants have been identified in a broad range of clinical and neurodevelopment phenotypes, presenting variable penetrance and expressivity [2-5]. They were recently identified in association with EAS, with about 20% of cases of LKS, ECSWS and RE cases presenting a *de novo* or inherited variant [2,6,7]. *GRIN2A* gene encodes a GluN2A protein, believed to be the most relevant GluN2 subunit of N-methyl-D-aspartate receptors (NMDAR) [7].

Sulthiame (STM), a sulfonamide derivative, was found to be efficient in focal and generalized epilepsies in 1960 [8,9]. It causes a global depression of intrinsic neuronal excitability, especially acting over NMDAR [8,9]. Although abandoned during the mid-1970s due to toxicity when used in combination with phenytoin, lately it has reemerged as monotherapy in SeLECTS or as an add-on therapy in EE-SWAS/DEE-SWAS [8-11]. It has also been found to be effective in the treatment of refractory epilepsies [8]. It is usually reported as a well-tolerated and safe anti-seizure drug, however, it may have adverse effects such as hyperpnea, paresthesias and anorexia [10].

Herein, we report a successful case of STM treatment in a female child with a diagnosis of EE-SWAS, who experienced adverse effects of corticosteroids and in whom a missense *GRIN2A* variant was identified.

## Case presentation

A previously healthy five-year-old female child, with normal growth and neurodevelopment and a family history of second- and third-degree maternal cousins with epilepsy during childhood, presented two de novo focal to bilateral tonic-clonic seizures during sleep. Her electroencephalogram (EEG) revealed right centrotemporal epileptiform activity intensified during sleep. She was initially diagnosed with SeLECTS and treated with VPA.

At six years of age, she had another focal to bilateral tonic-clonic seizure during sleep and started to present learning difficulties. Her teacher mentioned that she had been presenting important social interaction difficulties and was not able to acquire new skills. A second EEG revealed very broad right fronto-centro-temporal epileptiform activity, occasional during wakefulness and very abundant and with frequent generalization during about 80% of sleep, suggestive of EE-SWAS. Prednisolone was added to VPA during the subsequent 10 months (2 mg/kg/day for six months, followed by four months of progressive tapering). She recovered her baseline status and the subsequent EEG was normal.

**Figure 1:**
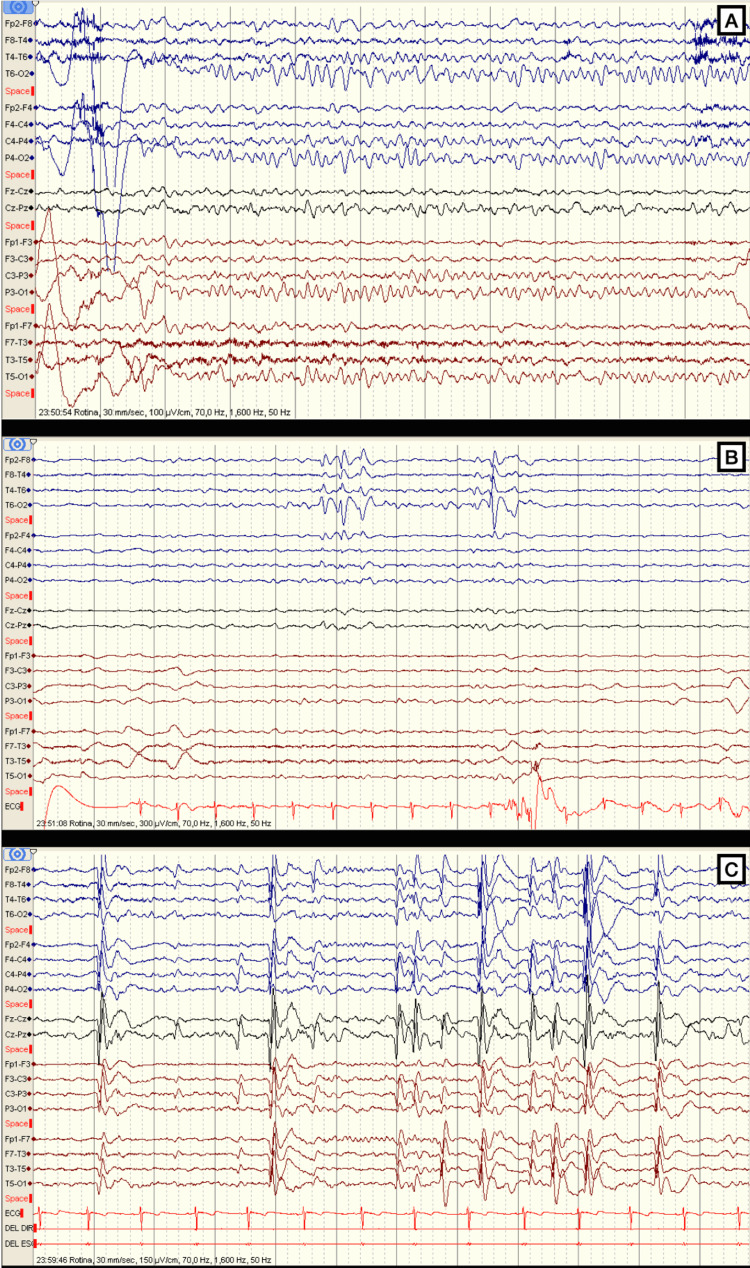
Patients’ video-EEG monitoring. A: Normal background rhythm in wakefulness. B: Spikes and spike-wave complexes of great amplitude and right fronto-centro-temporal location in wakefulness. C: Highly frequent epileptiform activity, sometimes with generalized bursts, and with greater amplitude, 500-1000uV, wider on the right, often at 1-2.5Hz, occupying 82.5% of non-rapid eye movement (REM) sleep.

One month after stopping prednisolone, language and learning problems were again noted, namely expressive aphasia, articulation errors, slurred speech, slowness and difficulty in writing/drawing and recognizing letters/words and difficulty in memorizing and acquiring new knowledge. A Wechsler Intelligence Scale for Children (WISC) assessment revealed a verbal intelligence quotient (IQ) of 89, a nonverbal IQ of 77, and a full-scale IQ of 79. Working memory index was evaluated through the Rey-Osterrieth Complex Figure (ROCF) test, where she copied a very disorganized figure, with identification of few original figure elements. Memory drawing was very primitive, without recognition of any element. Brain magnetic resonance imaging (MRI) was normal. Video-EEG monitoring identified near-continuous diffuse spike-and-wave complexes during sleep (Figure 1). Prednisolone was restarted (2 mg/kg/day), with significant clinical and electrographic improvement.

After a 12-month period of continuous prednisolone, the patient continued to show cognitive/behavioral improvement, without new seizures. However, adverse effects of corticosteroids were noted, including significant weight gain, cushingoid appearance and dyslipidemia with major psychosocial implications, namely bullying, leading to prednisolone suspension and VPA monotherapy maintenance.

Eight months later, she presented a new focal to bilateral tonic-clonic seizure during sleep and displayed increased language difficulties. A new EEG confirmed the recurrence of near-continuous diffuse spike-and-wave complexes during sleep. By this time, Sanger sequencing analysis revealed a missense variant of exon 14 of the GRIN2A gene, C.3228C>A (p.Asn1076Lys), classified as benign or probably benign. According to Mutation Taster and PolyPhen-2 bioinformatic analysis (tools used for prediction of the possible impact of an amino acid substitution on the structure and function of proteins) this was a predicted deleterious variant. STM (5 mg/kg/day) was added to VPA treatment (35 mg/kg/day).

The patient displayed important behavioral and cognitive improvement, showing residual articulation errors and slight slowness in writing. Also, she did not have any additional clinical seizures or diffuse spike-and-wave complexes during sleep recurrence. During the last four years, she has remained on the same therapy and none of the adverse effects of STM have been reported.

Both her mother and her younger sister have the same GRIN2A variant. Her mother has a history of learning difficulties during childhood, without known clinical seizures. Her seven-year-old sister remains healthy, presenting adequate academic performance. Her clinical follow-up will be maintained to monitor the development of possible future symptoms. Genetic testing of the maternal relatives with a history of epilepsy is not available.

## Discussion

EE-SWAS/DEE-SWAS evolution and response to treatment remain globally unpredictable and unsatisfactory, as reflected in our case [5]. We reported the case of a child diagnosed with EE-SWAS, with an identified GRIN2A missense variant, who experienced adverse effects of corticosteroids and abrupt language, learning and social regression with recurrence after their suspension. In this child, STM was used as an effective alternative. In this case, EE-SWAS was diagnosed in a pre-existing normal development child after the recognition of cardinal symptoms (cognitive, language, behavioral, motor and social interaction regression), temporally related to identification of marked activation of epileptiform abnormalities in sleep [1].

Until now, traditional EE-SWAS/DEE-SWAS treatment strategies have relied on the administration of various mono/polytherapy anti-seizure medications coupled with high-dose corticosteroids [5]. However, particularly in the pediatric population, corticosteroids comprise significant inherent and possibly irreversible adverse effects. In our case, besides organic consequences (weight gain, cushingoid appearance and dyslipidemia), the patient had major psychosocial implications, implying a need for a different therapeutical approach.

NMADR are cationic channels that are gated by glutamate, the most relevant excitatory neurotransmitter within the central nervous system [5,7]. Each consists of a heterotetrameric ionotropic complex, composed of two obligatory GluN1 subunits and two additional GluN2 or GluN3 subunits [5,7]. There are four different GluN2 subtypes (A-D), with GluN2A being considered the most important during childhood and adulthood [7]. The latter is encoded by the GRIN2A gene, which is located at chromosome 16 and consists of 14 exons [7].

GRIN2A gene variants represent, by far, the most reported genetic identified cause of EAS, especially in the most severe end of the spectrum, as in our patient [7,12]. The identification of these variants represented an important initial step for understanding their pathogenic mechanisms and testing treatment options. It led to efforts on trying to understand the impact of different GRIN2A variants on resultant mutant GluN2A and consequent final impact on NMDARs variable functioning [5,12]. Since NMDAR-mediated signaling is involved in development, plasticity, learning, memory and high cognitive functions, its dysfunction is seen in different neurodevelopmental disorders [5].

The described GRIN2A variant is categorized as benign or likely benign. However, variable penetrance and expression have been associated with GRIN2A variants, resulting in heterogeneous clinical and neurodevelopment phenotypes [2-5]. The same variant but different associated phenotypes were detected in our patient, her mother and her sister [2-5]. Although this variant is not classified as pathogenic, given the clinical evolution of the reported patient, the remarkable maternal family history of epilepsy, and the identification of the same variant in her mother, who also has a history of learning difficulties, we believe that this variant played a role in the development of these familial phenotypes. Also, according to Mutation Taster and PolyPhen-2 bioinformatic analysis, this was a predicted deleterious variant. Genetic testing of the maternal relatives with a history of epilepsy would have been useful for clarification but was not available. The follow-up of the younger sister may also play a role in elucidation.

STM acts through central carbonic anhydrase inhibition. It causes an extracellular proton concentration rise, which inhibits inward currents mediated by NMDAR and calcium currents through voltage-gated channels, consequently reducing neuronal excitatory function [9]. It also exerts a sodium channel-blocking effect in isolated hippocampal neurons, resulting in degradation of repetitive action potential generation [9]. Considering STM’s global intrinsic neuronal excitability depression mediated by NMDARs, it might present as a particularly interesting therapy to target EE-SWAS/DEE-SWAS cases associated with GRIN2A variants causing NMDAR gain-of-function. Although genotype-phenotype correlation remains elusive, some GRIN2A missense variants were previously reported as being associated with NMDAR gain-of-function [4].

It is difficult to establish a treatment strategy for EE-SWAS because there is no consensus about first-line therapy [13-16]. The usual treatment strategy involves the use of high-dose corticosteroids, anti-seizure drugs, and/or a ketogenic diet [13-16]. Valproic acid is one of the most commonly used anti-seizure drugs, but other options include levetiracetam, clobazam, topiramate, zonisamide, lacosamide, sulthiame, among others [13-16]. Intravenous immunoglobulins, adrenocorticotropic hormone or surgery are other treatment options [13-16].

In our patient, the recurrence of psychomotor regression, clinical seizures and temporally associated diffuse spike-and-wave complexes during sleep pattern, combined with corticosteroids' adverse effects, led to efforts in trying to find an alternative with better tolerability and response. Despite being currently classified as non-pathogenic and no functional study has been performed, the identification of a GRIN2A missense variant and NMDAR-related pathogenesis in EE-SWAS/DEE-SWAS led us to use the STM. This allowed for the prednisolone suspension and the patient's remarkable improvement and stability over the last few years. Furthermore, there were no adverse effects reported. This supports the possibility of functional GRIN2A variant studies becoming a future tool for designing and testing different treatment options, directed to the underlying genetic and pathophysiological mechanism. This may enable individualized interventions, possibly preventing most GRIN2A-related serious consequences.

## Conclusions

EE-SWAS/DEE-SWAS evolution and treatment success remain globally unpredictable. STM appeared to be an effective and safe therapy in this recurrent EE-SWAS patient, who presented a *GRIN2A* missense variant and adverse effects of corticosteroids. Functional *GRIN2A* variant studies might present as a future tool for designing and testing different and individualized treatment options in EE-SWAS/DEE-SWAS cases.
